# Unvalidated efficacy and significant risks hinder clinical use of deep cervical lymphatic-venous anastomosis for Alzheimer’s disease

**DOI:** 10.3389/fnagi.2025.1671741

**Published:** 2025-11-26

**Authors:** Rui Zhang, Ying Ying Liu, Xun Xia, Xinjun Li

**Affiliations:** 1 Department of Neurosurgery, School of Clinical Medicine and The First Affiliated Hospital of Chengdu Medical College, Chengdu, China; 2 Department of Neurology, Deyang People’s Hospital, Deyang, Sichuan, China; 3 Department of Neurosurgery, Huizhou Third People’s Hospital, Guangzhou Medical University, Huizhou, Guangdong, China

**Keywords:** Alzheimer’s disease, lymphatic-venous anastomosis, glial lymphatic system, meningeal lymphatic vessels, evidence-based medicine, medical ethics

## Abstract

Alzheimer’s disease (AD) is a neurodegenerative disorder characterized by cognitive decline and the pathological accumulation of amyloid-beta (Aβ) plaques and tau tangles. Recent studies suggest that dysfunction of the cerebral lymphatic clearance system may contribute to the progression of AD. This review critically examines the potential of deep cervical lymphatic-venous anastomosis (LVA) as a treatment for enhancing brain protein clearance and reducing cognitive decline in AD patients. Although animal models indicate that improving lymphatic drainage could facilitate Aβ clearance, clinical evidence is still insufficient. Current studies often have small sample sizes, short follow-up periods, and methodological weaknesses. Despite preliminary reports of cognitive improvements in small-scale clinical trials, the efficacy of LVA remains unproven, making widespread clinical adoption premature. Ethical concerns and technical challenges also pose significant barriers to clinical implementation. Rigorous randomized controlled trials (RCTs) are necessary to assess the long-term safety and efficacy of LVA for treating AD. Furthermore, the establishment of clear ethical and regulatory frameworks is essential before clinical use.

## 1 Introduction

Alzheimer’s disease (AD) is the most prevalent neurodegenerative disorder affecting the central nervous system (CNS). It is characterized by progressive cognitive deterioration, behavioral abnormalities, and severe impairment of activities of daily living (Anonymous, 2024). According to the International Alzheimer’s Association, nearly 50 million people worldwide were living with dementia in 2018. This number may double by 2050, reaching a prevalence of approximately 10% among adults aged over 65 years (Scheltens et al., 2021). Current therapeutic strategies primarily involve pharmaceutical agents, including cholinesterase inhibitors and N-methyl-D-aspartate (NMDA) receptor antagonists. Although these drugs can temporarily alleviate symptoms, they cannot halt or reverse the progression of AD (Zhu et al., 2020), resulting in an increasing burden on patients’ families and healthcare systems.

The classic pathological hallmarks of AD include β-amyloid (Aβ) plaques and tau neurofibrillary tangles, which are long-standing therapeutic research targets (Rajmohan and Reddy, 2017). Since the identification of the lymphatic system in the 17th century, the CNS was believed to lack functional lymphatic structures (Weller et al., 2009), leaving unresolved the mechanism for metabolic waste clearance in the brain. In 2012, Nedergaard’s team identified the glymphatic system, a brain-specific clearance pathway analogous to the peripheral lymphatic system, which facilitates waste removal through cerebrospinal fluid (CSF) and interstitial fluid exchange (Iliff et al., 2012). In 2015, Louveau and Aspelund’s teams discovered that meningeal lymphatic vessels (MLVs) connect to deep cervical lymph nodes (dcLNs), forming the primary lymphatic drainage pathway from the CNS to the periphery (Louveau et al., 2015). These findings led to the hypothesis that dysfunction of cerebral lymphatic clearance might drive AD pathogenesis (Das Neves et al., 2021; Jessen et al., 2015). Although this theory lacks adequate clinical validation, its identified anatomical targets have attracted considerable surgical interest.

Lymph-ovenous anastomosis (LVA) was initially developed as an ultramicrosurgical technique for peripheral lymphedema. Recently, some researchers have suggested LVA as a potential AD treatment. They hypothesize that reconstructing the cerebral cervical lymphatic drainage pathway may enhance intracerebral clearance of pathological proteins. Initial animal studies (Yao et al., 2018) indicate that improving MLV drainage could facilitate Aβ clearance and partially mitigate cognitive impairment. A preliminary clinical case report describing temporary postoperative cognitive improvement was subsequently recognized among the “Seven Advances in Microsurgery for 2022” (Lu et al., 2022). These early findings have raised cautious optimism regarding AD treatment.

However, such optimism is accompanied by concerns. Following the release of several exploratory studies, some medical institutions have rapidly adopted clinical applications of LVA. Public data indicate that, as of 2025, 382 hospitals across 32 provinces/municipalities in China offer LVA for AD (excluding Tibet and Hong Kong). Many applications rely on preliminary results from small-sample, short-term, non-randomized studies, yet some reports prematurely describe these outcomes as “breakthroughs.” This rapid clinical translation poses significant risks due to insufficient scientific validation. It might mislead research directions, ethically compromise vulnerable populations, and clinically expose patients to unrecognized hazards. Given the unproven status of the cerebral lymphatic clearance theory and insufficient empirical evidence of LVA’s efficacy and safety in AD, it is critical to address key controversies, identify factors driving rapid adoption, and urge adherence to scientific rigor. This cautious approach will ensure adequate evidence guides the future development of this technology.

### 1.1 Literature retrieval methods

This study is a narrative review. The literature retrieval strategy was as follows: keywords including “AD,” “lymphatic-venous anastomosis,” “meningeal lymphatic vessels,” “supermicrosurgery,” “glymphatic system,” and “clinical trial” were used to search PubMed, Web of Science, Embase, Cochrane Library, CNKI, and Wanfang databases, covering the period from 2012 (the year the glymphatic system was discovered) to June 2025. Inclusion criteria comprised original studies (preclinical animal experiments, clinical case reports, and observational studies), systematic reviews, and meta-analyses. Exclusion criteria included conference abstracts, promotional materials, and duplicate publications. A total of 76 articles were included, consisting of 17 preclinical studies, 19 clinical studies, 36 reviews or commentaries, and 4 other publications (including a thesis, ethical guideline, historical commentary, and expert consensus).

## 2 Theoretical basis: cerebral lymphatic clearance system and AD pathogenesis

The theoretical rationale for LVA in AD lies in targeting dysfunction of the cerebral lymphatic drainage system. Impaired cerebrospinal fluid (CSF) circulation and lymphatic drainage may contribute to AD pathology through multiple mechanisms:

### 2.1 Cerebrospinal fluid circulation impairment and pathological initiation of AD

Abnormal CSF circulation is considered an early event in the pathological cascade of AD. Reduced CSF production appears in the early stages of the disease, with secretion levels potentially declining to 50% of normal by the middle to late stages. This reduction is directly linked to impaired mitochondrial function in choroid plexus epithelial cells (Constantinides et al., 2023; Kant et al., 2018). Concurrently, CSF composition undergoes marked changes: a decreased Aβ42/Aβ40 ratio reflects the influence of intracerebral Aβ deposition on the brain microenvironment (De Leon et al., 2017), while reduced CSF flow velocity in the lateral ventricles and superior nasal concha regions correlates negatively with Aβ plaque burden (Weller et al., 2008). These findings suggest that impaired CSF clearance may be a key driver of Aβ aggregation.

### 2.2 Structural and functional abnormalities of the perivascular space

The PVS serves as a crucial interface for CSF–interstitial fluid (ISF) exchange between the CSF and brain tissue (Chen S. et al., 2025; Wardlaw et al., 2020). In AD patients, basement membrane alterations, vascular smooth muscle degeneration, and Aβ deposition disrupt the structural integrity of the PVS. These changes reduce its effective cross-sectional area, weakening convective clearance driven by arterial pulsations (Silva et al., 2021). This establishes a vicious cycle of “obstruction–clearance impairment–accelerated deposition,” exacerbating waste accumulation in the brain.

### 2.3 Deterioration of the glymphatic system

Impaired glymphatic function further reduces clearance efficiency. The polarized distribution of aquaporin-4 (AQP4) on astrocytic endfeet is disrupted in AD, directly diminishing interstitial fluid flow and promoting tau and Aβ accumulation (Harrison et al., 2020; Tarasoff-Conway et al., 2015; Yamada and Iwatsubo, 2024). Animal studies confirm a direct correlation between tau aggregation and reduced glymphatic clearance capacity (Da Mesquita et al., 2018a; Louveau et al., 2015).

### 2.4 Craniocervical drainage axis degeneration

The craniocervical drainage axis, composed of meningeal lymphatic vessels (MLVs) and deep cervical lymph nodes (dcLNs), undergoes age-related degeneration, including reduced MLV diameter, density, and function (Wang et al., 2019; Yao et al., 2018). In APP/PS1 mice, dcLN ligation impairs lymphatic drainage, disrupts AQP4 polarization, and induces cognitive decline (Da Mesquita et al., 2018b). Conversely, VEGF-C–mediated MLV neogenesis enhances Aβ clearance and improves cognition (Olate-Briones et al., 2022). These findings suggest that lymphatic drainage dysfunction is a key pathological mechanism in AD and a potential therapeutic target.

### 2.5 Other potential mechanisms

#### 2.5.1 Neuroinflammation and immune dysregulation

MLV dysfunction impairs communication between the CNS and peripheral immune system, leading to inadequate antigen presentation and immune cell retention in the brain. This triggers pro-inflammatory cytokine release and microglial activation (Kaur et al., 2024; Lu et al., 2022; Valiukas et al., 2025; Xie et al., 2024), forming a harmful cycle of “impaired drainage → immune retention → inflammatory activation” that accelerates AD pathogenesis.

#### 2.5.2 Impact on vascular compliance

Abnormal cervical lymphatic drainage may also affect cerebral hemodynamics. AD patients often exhibit reduced vascular elasticity. Kyrtsos and Baras, using a multi-compartment mathematical model (Kyrtsos and Baras, 2015), demonstrated that doubling vascular stiffness significantly increases Aβ40 and Aβ42 deposition in brain parenchyma. This occurs because vascular rigidity reduces ISF convective flow, disrupting Aβ transport from the parenchyma to the PVS.

#### 2.5.3 Lymphatic–venous interactions

The perivenous clearance pathway of the lymphoid-like system serves as a key route for brain metabolic waste removal, depending on venous structural integrity and drainage dynamics. According to the mechanism proposed by Iliff et al. (Iliff et al., 2012), this pathway uses perivenous spaces as conduits and relies on the arteriovenous hydrostatic gradient generated by arterial pulsations to drive ISF convection. Clinical studies by Pardo et al. (2025) in AD patients demonstrate that venous structural and functional abnormalities directly impair clearance efficiency. Venous lumen stenosis reduces drainage capacity, disrupting the pressure gradient essential for lymphatic-like function and slowing metabolic waste clearance.

**FIGURE 1:**
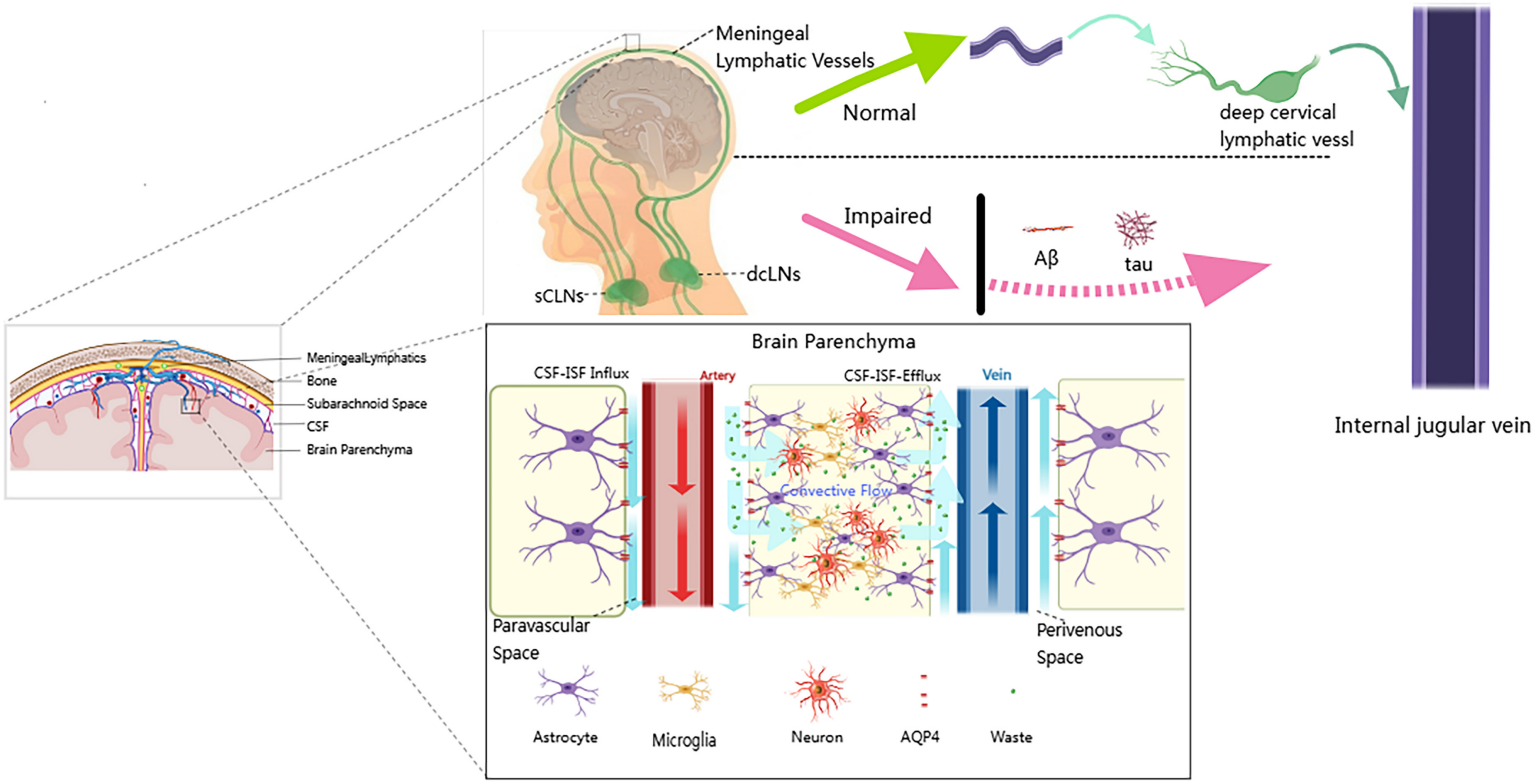
Schematic comparison of physiological and pathological cerebrospinal fluid-lymphatic clearance systems. Upper panel (Normal): Depicts the efficient waste clearance pathway in a healthy brain. Cerebrospinal and interstitial fluids (CSF–ISF) enter the brain parenchyma through perivascular spaces. This convective flow is driven by the polarized distribution of AQP4 water channels on astrocytic endfeet. Metabolic waste is cleared from the extracellular environment of neurons and microglia and transported via MLVs to the dCLNs for drainage into the systemic circulation through the internal jugular vein. Lower panel (Impaired): Shows the dysfunctional clearance system in AD. Major impairments include: (1) loss of AQP4 polarization on astrocytes; (2) obstruction of perivascular spaces by pathological protein deposits; and (3) degeneration of MLVs. These abnormalities result in waste clearance failure and the accumulation of pathological proteins, such as Aβ and tau, within the brain parenchyma, driving neuroinflammation and neuronal injury. Image created by the authors using MedPeer.

Collectively, these multifaceted impairments in the cerebral clearance system are illustrated in Figure 1, contrasting the physiological and pathological states in AD. Under normal conditions (upper panel), CSF efficiently removes metabolic waste via the PVS, facilitated by polarized AQP4 channels, and drains through MLVs and dcLNs. In AD (lower panel), this system fails, with loss of AQP4 polarization, PVS obstruction, and MLV degeneration, resulting in Aβ and tau accumulation. This schematic illustrates the pathophysiological rationale underlying LVA surgery.

## 3 Preclinical model evidence: findings and limitations from animal studies

Preclinical research provides preliminary support for the theoretical basis of LVA treatment in AD. However, results vary among models, and interspecies differences limit clinical translation. According to the Oxford Center for Evidence-Based Medicine (OCEBM) evidence grading system (where animal studies are classified as Level V, the lowest), major preclinical findings are summarized in Table 1, together with methodological characteristics and limitations.

**TABLE 1:** Evidence grading and methodological analysis of LVA treatment in AD-related preclinical studies.

Research team/year	Animal model	Intervention	Experimental duration	Key results	Evidence level (OCEBM)	Limitations
Yao et al. (2018)	APP/PS1 transgenic mice (6 months old)	VEGF-C-mediated meningeal lymphatic vessel regeneration	2 weeks (Cannula implantation for 4 days, followed by 4 rhVEGF-C injections every 2 days; samples collected 7 days after the last injection)	Increased density of meningeal lymphatic vessels, improved Aβ clearance efficiency, and enhanced spatial cognitive scores	V	Evaluated only short-term effects (4 weeks); aging process not simulated
Wang et al. (2019)	APP/PS1 transgenic mice (8 months old)	Ligation of deep cervical lymph nodes	1 month (behavioral testing and pathological analysis conducted 1 month post-ligation surgery)	25% decrease in glymphatic drainage efficiency, 20% increase in Aβ deposition	V	“Injury model” mechanism differs from “repair model” of LVA
Da Mesquita et al. (2018b)	Naturally aged mice (24 months old) + AD model mice (12 months old)	Morphological analysis of meningeal lymphatic vessels	Single time-point morphological analysis (no intervention period)	40% reduction in MLV diameter in aged mice; 35% decrease in MLV density in AD model mice	V	Correlational study without intervention
Harrison et al. (2020)	rTg4510 tau transgenic mice (5 months old)	Glymphatic system function monitoring	Single measurement of glymphatic function (no prolonged intervention)	40% decrease in tau clearance rate, disordered AQP4 polarization	V	No direct intervention on lymphovenous pathways

APP/PS1, Amyloid Precursor Protein/Presenilin 1; VEGF-C, Vascular Endothelial Growth Factor-C; rhVEGF-C, Recombinant Human Vascular Endothelial Growth Factor-C; Aβ, Amyloid β-Protein; LVA, Deep Cervical Lymphatic-Venous Anastomosis; MLV, Meningeal Lymphatic Vessels; AQP4, Aquaporin 4.

### 3.1 Key findings from preclinical studies

1. Positive Effects of Meningeal Lymphatic Vessel Regeneration: Studies confirm that intravitreal VEGF-C injection promotes MLV regeneration and function. This significantly improves Aβ clearance (reduced CSF Aβ42; increased lymph node Aβ) and cognitive performance (Yao et al., 2018). This suggests that enhancing meningeal lymphatic drainage could represent an effective AD treatment strategy.

2. Importance of Deep Cervical Lymph Node Function: Ligation of deep cervical lymph nodes exacerbates AD-like pathology (increased Aβ deposition and cognitive decline) in APP/PS1 mice (Wang et al., 2019). This finding confirms the importance of an unobstructed craniocervical drainage axis for cerebral waste removal, indirectly supporting the hypothesis behind LVA (restoration of cervical lymphatic drainage).

3. Age-Related Degeneration of the Lymphatic System: Natural aging leads to significant structural and functional deterioration (reduced MLV diameter and density) (Da Mesquita et al., 2018b). This degeneration overlaps with AD pathology, indicating LVA may hold greater therapeutic promise in elderly AD patients. However, current animal models do not fully replicate human aging processes.

### 3.2 Limitations of preclinical studies

#### 3.2.1 Species differences as an insurmountable barrier

Significant differences in lymphatic system structure and function between animals and humans considerably hinder LVA’s clinical translation. In humans, meningeal lymphatic vessels (mLVs) exhibit a linear arrangement along the sagittal sinus, complex anatomy, and substantial individual variability. Specific drainage routes to deep cervical lymph nodes (dcLNs) require validation by dynamic imaging and pathological studies (Zhou et al., 2020). Conversely, rodent mLVs have simpler structures with notably different drainage patterns. Mice possess fewer dcLNs with limited drainage, whereas humans have around 450 cervical lymph nodes (Haley, 2017), forming a highly complex and multi-layered network. These nodes intricately interact with retropharyngeal lymph, peripheral lymph surrounding the internal jugular vein, and intracranial lymph (Yağmurlu et al., 2020). Procedures like dcLN ligation in animal studies might induce non-physiological compensatory mechanisms that obscure pathological findings (Wang et al., 2019). Although certain aquatic species possess advanced mLVs, substantial evolutionary divergence from humans limits their anatomical relevance (Jackson et al., 2025). Consequently, inherent biological differences substantially increase uncertainty in clinical translation.

#### 3.2.2 Absence of a human aging model

Current animal models do not accurately replicate the progressive decline of the cerebral lymphatic clearance system associated with human aging. Alzheimer’s disease predominantly affects older adults. Aging in humans involves structural degeneration of meningeal lymphatic vessels (thickened lumen, reduced diameter) (Jaffe et al., 2019; Plog and Nedergaard, 2018) and functional impairment of dcLNs (reduced filtration and immune response) (Gao et al., 2025). This leads to decreased cerebral waste clearance efficiency. However, commonly used Alzheimer’s disease models (e.g., 5xFAD) primarily replicate Aβ pathology, lack typical neurofibrillary tangles (NFTs), and overly rely on early-onset genetic mutations. Thus, they inadequately model late-onset AD’s chronic progression and complex pathogenesis (Oblak et al., 2021). Immune senescence in aging is characterized by chronic low-grade inflammation and glial dysfunction (Merighi et al., 2022). Its potential impact on lymphatic drainage and waste removal remains poorly studied, and current models fail to reproduce this complex phenotype (Peplow and Martinez, 2019). Moreover, aging of the human lymphatic system involves genetic, environmental, and comorbid factors, resulting in considerable individual variabilityy (Liu et al., 2023). Animal models cannot replicate this complexity, complicating predictions about lymphatic augmentation efficacy in elderly patients.

#### 3.2.3 Differences between LVA simulation and clinical surgery

Preclinical studies often utilize interventions such as meningeal lymphatic vessel regeneration (VEGF-C) or cervical lymph node ligation (simulating functional impairment). Actual surgical anastomosis techniques (such as microsurgical anastomosis of ∼0.5 mm vessels) are difficult to replicate in animal models (Thamm et al., 2024). Therefore, assessing the surgical feasibility and long-term effectiveness of LVA remains challenging.

## 4 Clinical evidence: characteristics of existing studies and evidence-based deficiencies

Clinical evidence supporting LVA treatment for AD remains in the early exploratory phase, with very limited evidence (OCEBM Levels IV–V). To systematically assess the current research, existing evidence is summarized in two tables: Table 2 (Study Design and Basic Information) lists key parameters such as study centers, study designs, sample sizes, and evidence grades. Table 3 (Study Outcomes and Limitations) focuses on postoperative efficacy measures, follow-up outcomes, and reported limitations.

**TABLE 2:** Basic information and design of studies at each center.

Clinical center	Study design type	Sample size	Key inclusion criteria (example)	Surgical protocol	OCEBM grade
Hangzhou Qiushi Hospital (Lu et al., 2022)	Single-Case Exploratory Study	1 case	Age 84, severe AD, ineffective medical treatment	3D Endoscope-Guided Deep Cervical Lymphatic-Venous Anastomosis	5
Shanghai Ninth People’s Hospital (Li X. et al., 2024)	Prospective Single-Arm Observational Study	6 cases	Age ∼70, severe AD (Montreal Cognitive Assessment (MoCA): 5 points)	Cervical Shunting to Unclog Cerebral Lymphatic Systems (CSULS)	4
Shanghai Ninth People’s Hospital (Li et al., 2025)	Prospective Single-Arm Observational Study	7 cases	Mean age 65, confirmed AD	Cervical Level II/III Lymphatic Vessel-Lymph Node-Venous Anastomosis	4
Plastic Surgery Hospital, Chinese Academy of Medical Sciences (Gan et al., 2025)	Prospective Single-Arm Observational Study	4 cases	Ages 58–79, moderate-to-severe AD	High-Frequency Ultrasound-Guided Deep Cervical Lymph Node-Venous Anastomosis	4
Southwest Hospital, Army Medical University (Chen J. Y. et al., 2025)	Prospective Single-Arm Exploratory Study	26 cases	Ages 54–77, meeting ATN biomarker criteria	Modified Lymphatic Flap-Venous Anastomosis	4

AD, Alzheimer’s Disease; MoCA, Montreal Cognitive Assessment; CSULS, Cervical Shunting to Unclog Cerebral Lymphatic Systems; ATN, Amyloid-Tau-Neurodegeneration; OCEBM, Oxford Center for Evidence-Based Medicine.

**TABLE 3:** Research findings and limitations across centers.

Clinical center	Primary outcome measures	Postoperative follow-up and key results	Limitations
Hangzhou Qiushi Hospital (Lu et al., 2022)	Neuropsychological scales: MMSE, MoCA, NPI, ADCS-ADL; Imaging: Brain MRI; Safety: Surgical complications	180-day follow-up: MMSE increased from 3 to 18; MoCA from 2 to 13,NPI complete remission, ADCS-ADL normalized; MRI (7 months post-op): Ventricular volume reduced, sulcal shallowing; No surgery-related complications	Single-case report, lacks generalizability; No long-term follow-up data; Placebo effect not excluded; Persistence of cognitive improvement unclear
Shanghai Ninth People’s Hospital (Li W. et al., 2024)	Neuropsychological scales: MoCA, CDR-SB; Imaging: PET (tau/Aβ deposition, cerebral glucose metabolism)	5-week follow-up: MoCA ncreased from 5 to 7, CDR-SB decreased 10 to 8; PET: Reduced tau protein deposition, increased temporal lobe glucose metabolism; No complications (bleeding/nerve injury)	Small sample size; Short follow-up duration; Lack of control group; Potential caregiver observation bias; Long-term safety unclear
Shanghai Ninth People’s Hospital (Li et al., 2025)	Neuropsychological scales: MMSE, MoCA, NPI; Imaging: PET (whole-brain Aβ quantification); Neck ultrasound (internal jugular vein patency)	1-month follow-up: MMSE improved 0 points, MoCA improved 0 points, NPI improved 9 points; PET: Reduced whole-brain Aβ; Ultrasound: Internal jugular veins patent, no thrombosis	Short follow-up duration; No significant cognitive improvement; No randomized control; Results reliant on caregiver reports; No long-term PET data
Chinese Academy of Medical Sciences & Peking Union Medical College, Plastic Surgery Hospital (Gan et al., 2025)	Neuropsychological scales: MMSE, ADAS-Cog, NPI, ADCS-ADL; Safety: Surgical complications; Lab: Coagulation function, inflammation markers	1-month follow-up: MMSE avg. increase 0.8 points, ADAS-Cog decreased 1.0-11.3 points (2 cases decreased > 5 points), NPI/ADCS-ADL improvement rate 75%; 3 cases mild local swelling/skin paresthesia, resolved within 2 weeks; No significant lab changes	Limited sample size; Very short follow-up; Mild cognitive improvement; No long-term safety/efficacy data; Not compared with standard AD treatment
Southwest Hospital, Army Medical University (Chen J. Y. et al., 2025)	Neuropsychological scales: MMSE, MoCA, NPI; Lab: CSF biomarkers (Aβ42, Aβ40, Tau); Safety: Surgical complications; Imaging: PET/MR	1-month follow-up: MMSE improved 3→5 (orientation, memory registration), MoCA (improved in 15%), NPI (decreased in 42%), neither statistically significant; CSF biomarkers trended downward; 2 patients experienced temporary postoperative arm elevation difficulty	Single-arm design, no control; Short follow-up, lacks mid-term cognitive & pathological biomarker dynamic data; PET/MR imaging quantitative results incomplete; Aβ/tau clearance efficiency not verified

### 4.1 Common evidence-based deficiencies in clinical research

#### 4.1.1 Insufficient sample size and follow-up duration

Four out of five studies (Table 2) had sample sizes of seven cases or fewer; only the study from Southwest Hospital of Army Medical University enrolled 26 cases. Individual variability and placebo effects cannot be excluded. The longest follow-up was 180 days (6 months), while most were ≤ 1 month. Because AD is a chronic progressive disease, short-term follow-up fails to assess sustained efficacy or delayed complications (e.g., anastomotic obstruction or cognitive rebound).

#### 4.1.2 Research design limitations

None of the studies used randomized controlled designs; all lacked sham surgery or standard treatment control groups. Thus, distinguishing between surgical effects and natural fluctuations in disease progression is impossible. No studies implemented blinding (surgeons, evaluators, and patient families were aware of treatments), potentially causing assessment bias. Cognitive function in AD patients naturally fluctuates in the short term18 (Ávila-Villanueva et al., 2022), and the placebo effect associated with surgical intervention may further amplify these fluctuations (Chen et al., 2022).

#### 4.1.3 Comorbidity confounding factors

Potential comorbid idiopathic normal pressure hydrocephalus (iNPH) was inadequately ruled out (Gallia et al., 2006). About 26% of iNPH patients exhibit AD-related pathology, with overlapping biomarkers (e.g., CSF Aβ42) (Mazzeo et al., 2022). Moreover, iNPH patients may show cognitive improvement following CSF shunt surgery (Kazui et al., 2016). In the initial LVA case report (Lu et al., 2022), cognitive improvement in patients with coexisting iNPH might result from enhanced CSF dynamics rather than improved clearance of AD proteins, possibly causing misinterpretation of therapeutic effects.

#### 4.1.4 Heterogeneity in outcome measures

Studies used inconsistent cognitive assessment scales (MMSE, MoCA, ADAS-Cog, CDR-SB) and imaging methods (MRI, PET), with no unified efficacy standards. Some studies relied on subjective scales (e.g., MMSE) without integrating objective biomarkers (e.g., CSF tau levels), limiting credibility.

These evidence-based deficiencies in LVA studies are not isolated. Other invasive neurosurgical interventions for AD, such as deep brain stimulation (DBS), face similar global clinical challenges, including low-level evidence (primarily OCEBM IV–V), biased study designs (e.g., lack of blinding, selective reporting), and ethical controversies (Bittlinger and Müller, 2018).

## 5 Technical barriers: practical challenges in surgical implementation and efficacy validation

The technical complexity of LVA therapy for AD significantly exceeds that of lymphoedema treatment. Existing technologies have not resolved key challenges related to precise anastomosis and quantitative evaluation of therapeutic efficacy.

### 5.1 Technical challenges in surgical procedures

Difficulty in Target Localization: Deep cervical lymphatic vessels typically measure less than 0.5 mm in diameter, with thin, translucent walls. Identification of functional lymphatic vessels usually depends on near-infrared fluorescence imaging (ICG) or intraoperative observation of lymphatic fluid flow (Thamm et al., 2024). However, ICG visualization depth is limited to superficial lymphatics (penetration < 1 cm) (Mito et al., 2022), insufficient for deep cervical lymphatics (>2 cm depth), thus increasing the risk of misidentifying surgical targets.

#### 5.1.1 Controversy over anastomosis techniques

End-to-end anastomosis preserves vascular wall integrity, significantly reducing risks and enhancing unidirectional lymphatic-to-venous flow (Onoda et al., 2023). This method requires precise alignment of vessel diameters. End-to-side anastomosis is simpler to perform and maintains shunt patency even with severe lymphatic valve loss, establishing a unidirectional pressure gradient following distal vein ligation (Kwon et al., 2022). However, this method increases thrombosis risk due to altered hemodynamics. Long-term patency remains uncertain for both techniques. Factors such as cervical movement, vascular pulsation, and postoperative fibrosis may cause anastomotic stenosis or occlusion. No available study data provide anastomotic patency outcomes beyond one year.

#### 5.1.2 Surgical risks in elderly patients

Elderly AD patients have unique physiological vulnerabilities, significantly increasing operative risks. Poor anesthetic tolerance, fragile vascular structures due to sclerosis, and deteriorating organ function are common in elderly patients, elevating complication risks (Di Nino et al., 2010). Frequent postoperative complications include pneumonia, lower-limb deep vein thrombosis, and transient delirium (Thillainadesan et al., 2022). Neck-specific complications, such as recurrent laryngeal nerve injury and internal jugular vein rupture, have not been reported but remain significant potential risks. Another subtle risk is that surgery may disrupt physiological lymphatic drainage, and indiscriminate intervention could cause drainage imbalance, exacerbating metabolic waste accumulation and accelerating disease progression.

### 5.2 Gaps in the efficacy assessment system

The lack of reliable tools for accurately evaluating the effect of LVA on the function of the craniocervical drainage axis poses a major obstacle to clinical translation. Current imaging techniques have significant limitations: indocyanine green lymphography (ICG) has inadequate penetration depth (Mo et al., 2024); magnetic resonance lymphography (MRL) lacks real-time functional evaluation (Pons et al., 2019); and microbubble contrast-enhanced ultrasound (CEUS) relies on adjacent veins and cannot assess lymphatic vessel integrity (Jang et al., 2022). None of these methods measure the rate of intracranial waste clearance through the lymphatic system. They also cannot directly link improved lymphatic outflow with cognitive changes.

Existing Alzheimer’s disease biomarkers, such as Aβ42 and tau proteins in cerebrospinal fluid (Jack et al., 2018), primarily indicate pathological accumulation rather than lymphatic clearance efficiency. Thus, they cannot directly validate lymphatic drainage function. Even if peripheral Aβ concentrations change after surgery, it remains unclear whether this is due to enhanced intracranial clearance or altered peripheral metabolism. Functional imaging (PET imaging) can detect brain Aβ and tau changes (Boccalini et al., 2025), but it cannot determine whether these changes reflect increased clearance or decreased production. Furthermore, the Diffusion Tensor Image Analysis Along the Perivascular Space (DTI-ALPS) index, an indirect marker of glymphatic function, currently lacks evidence directly correlating it with lymphatic drainage (Ren et al., 2025). These limitations impede the establishment of a clear causal pathway (“surgical intervention–drainage improvement–pathologic reduction–cognitive enhancement”). This leads to the clinical dilemma of being unable to confirm or deny the effectiveness of LVA, significantly increasing the risk of resource waste in large-scale clinical studies.

This critical bottleneck has stimulated global research efforts toward developing next-generation imaging technologies for precise, non-invasive, and quantitative evaluation of the craniocervical drainage axis. Recent technological advancements have addressed the limitations of existing clinical tools, providing promising directions for objectively measuring LVA’s physiological effects. For simultaneous visualization of structural and dynamic processes, a Chinese research team developed dual-contrast functional photoacoustic microscopy (DCF-PAM). This technology, for the first time, differentiated meningeal lymphatic vessels (MLVs) from parenchymal lymphatic-like pathways in vivo in mice (Yang et al., 2024). Research showed that lymphatic drainage volume through MLVs in AD model mice decreased by 70%. This method could potentially track hemodynamic changes in MLVs distal to lymphatic-vascular anastomosis, avoiding reliance on superficial lymphatic imaging. To elucidate the relationship between neural regulation and glymphatic function, researchers from the Massachusetts Institute of Technology (MIT) combined *in vivo* two-photon imaging with multisensory gamma stimulation. They identified a regulatory axis (“neural activity–arterial pulsation–glymphatic clearance”) (Murdock et al., 2024). This finding suggests that structural reconstruction (LVA) combined with functional enhancement (neuromodulation) might represent a synergistic therapeutic strategy. Their proposed method for quantifying glymphatic efflux dynamics offers a precise validation tool for determining whether LVA activates glymphatic function effectively. For non-invasive bedside monitoring, near-infrared II (NIR-II) imaging allows non-invasive transcranial assessment of glymphatic function in mice (Li W. et al., 2024). This technology could facilitate real-time bedside monitoring and dynamic tracking of drainage rates at critical postoperative intervals (e.g., 1 week, 1 month), enabling early detection of anastomotic dysfunction. Importantly, to quantify aging’s effect on surgical targets, a U.S.-Danish team used two-photon particle tracking technology. They discovered age-related functional decline in cervical lymphatic vessels (cLVs), characterized by reduced contraction frequency and slower flow velocity (Du et al., 2024). This finding highlights that if target cLVs themselves exhibit functional impairment, merely reconstructing the pathway may not achieve optimal outcomes. Once these technologies successfully transition to clinical practice, preoperative evaluation of cLV function could become essential for personalized treatment.

Currently, these technologies remain primarily at the preclinical stage. Therefore, validated and reliable assessment tools suitable for human LVA clinical trials have yet to be established, representing a future challenge that must be overcome.

## 6 Ethical regulatory challenges in the Chinese context and global commonality

The clinical application of LVA for AD treatment faces substantial ethical and regulatory challenges. Cognitive impairments inherent in AD compromise patients’ decision-making capacities. Patients with mild impairment may struggle to fully comprehend the long-term risks and prognosis associated with surgery (Gaubert and Chainay, 2021). Meanwhile, moderate-to-severe patients typically lack autonomous decision-making abilities and must rely on family members or legal representatives. The latter frequently experience significant pressures during decision-making (Dimech, 2019). Driven by a psychological motivation of “last hope,” families may underestimate surgical risks. Additionally, some institutions exaggerate efficacy and downplay risks in promotional materials, reducing informed consent to a mere formality and violating the principle of patient autonomy.

According to the “minimal risk” principle outlined in the Declaration of Helsinki (World Medical Association, 2013), LVA, as an invasive neck procedure, carries potential severe complications, placing its risks well beyond the “minimal risk” threshold. The ethical justification for labeling such high-risk surgery as a “breakthrough therapy” and applying it to vulnerable populations with impaired decision-making capacity is questionable. Particularly for severe dementia patients with very low MMSE scores, who essentially have lost autonomous decision-making abilities (Gilbert et al., 2022), even technically successful surgery offers limited improvement to their quality of life. Thus, profound ethical concerns exist about whether the procedure genuinely serves patients’ best interests.

The current regulatory environment exacerbates these risks. Unified ethical standards and regulatory frameworks for novel neurosurgical interventions remain absent. Regulatory practices vary significantly by region: while Europe and the United States typically require rigorous clinical trial approval processes, many Asian regions (including China) have comparatively lenient regulatory standards (Karlawish et al., 2005). These regulatory differences may encourage stakeholders to conduct procedures in regions with less stringent oversight to circumvent necessary review procedures. The high cost of these interventions provides substantial financial incentives for medical institutions. Some facilities classify LVA as a “routine treatment” rather than a clinical trial, bypassing essential ethical review processes and increasing potential risks. Overinterpretation of early-stage, data-limited “expert consensus” (Xie Q. P. et al., 2025) further blurs the boundary between scientific research and established clinical practice, potentially misleading primary care providers and patient populations.

The rapid proliferation of LVA for AD treatment is quantitatively illustrated by the surge in clinical trial registrations in China (Table 4). A public database search (Chinese Clinical Trial Registry, ChiCTR) shows over 20 new trials on LVA for AD registered in a short period (2024 to mid-2025). This trend, involving numerous prominent medical institutions nationwide, is driven by pressures related to academic evaluations and anxiety regarding innovation. The sheer volume of registered trials provides macro-level evidence of widespread clinical investigation despite the procedure’s unproven efficacy and uncertain safety profile, as detailed previously. This phenomenon is not isolated but reflects the broader global predicament faced by invasive neurosurgical interventions for dementia. A systematic review by Gilbert et al. (2022) revealed a significant upward trend in both the number and scale of invasive dementia trials worldwide (including deep brain stimulation, stem cell implantation, and gene therapy) between 2014 and 2020. Within this global context, protecting informed consent capacity and conducting rigorous risk-benefit assessments have emerged as central controversies (Gilbert et al., 2022). The review further identifies substantial heterogeneity in inclusion criteria across trials globally and widespread lack of transparency regarding ethical review processes, highlighting the absence of international consensus on research regulation.

**TABLE 4:** Registered clinical trials investigating deep cervical LVA for AD in China.

Registration number	Registration title	Research institution	Study type
ChiCTR2500105493	Study on the Brain Network Mechanism of Deep Cervical Lymphatic-Venous Anastomosis in the Treatment of Alzheimer’s Disease Based on fNIRS	General Hospital of the Central Theater Command of the Chinese People’s Liberation Army	Interventional Study
ChiCTR2500105306	Exploratory Study on Improving Brain Function in Alzheimer’s Disease by Cervical Lymphatic Vessel/Nodal-Venous Anastomosis	Ninth People’s Hospital Affiliated to Shanghai Jiao Tong University School of Medicine	Interventional Study
ChiCTR2500104671	Clinical Observational Study on Cervical Lymphatic Vessel/Nodal-Venous Anastomosis in the Treatment of AD-Induced Cognitive Impairment	Affiliated Hospital of Zunyi Medical University	Interventional Study
ChiCTR2500104509	Treatment of Alzheimer’s Disease with Deep Cervical Lymphatic-Venous Anastomosis (LVA)	Baotou Central Hospital	Interventional Study
ChiCTR2500104139	Treatment of Alzheimer’s Disease with Lymphatic-Venous Anastomosis	Peking University Shenzhen Hospital	Observational Study
ChiCTR2500102675	Study on the Effects of Deep Cervical Lymphatic-Venous Anastomosis on Biomarkers, Efficacy and Safety in Alzheimer’s Disease at Different Stages	Guiyang Second People’s Hospital	Interventional Study
ChiCTR2500102667	Study on Improving Cognitive Function in Alzheimer’s Disease Patients by Deep Cervical Lymphatic-Venous Anastomosis Combined with Drug Therapy versus Drug Therapy Alone	Affiliated Hospital of Guangdong Medical University	Interventional Study
ChiCTR2500101778	Study on the Safety, Feasibility and Efficacy of Deep Cervical LVA Surgery for the Treatment of Moderate to Severe AD	Changzhou Second People’s Hospital	Interventional Study
ChiCTR2500101642	Single-Center Prospective Clinical Study on Deep Cervical Lymphatic Vessel/Nodal-Venous Anastomosis in the Treatment of AD	First Affiliated Hospital of Xi’an Jiaotong University	Interventional Study
ChiCTR2500101614	Exploratory Study on Deep Cervical LVA in the Treatment of Advanced Alzheimer’s Disease	Peking University School and Hospital of Stomatology	Interventional Study
ChiCTR2500099855	Study on the Efficacy and Safety of Deep Cervical LVA Combined with Lymphatic System Remodeling in the Treatment of Moderate to Severe AD	China-Japan Union Hospital of Jilin University	Interventional Study
ChiCTR2500098639	Exploratory Study on Improving Brain Function in Alzheimer’s Disease by Cervical Lymphatic Vessel/Nodal-Venous Anastomosis	Second Hospital of Jilin University	Interventional Study
ChiCTR2500098356	Single-Center, Prospective Self-Controlled Clinical Study on Deep Cervical LVA in Improving Neurological Function in Alzheimer’s Disease Patients	First Medical Center of Chinese PLA General Hospital	Interventional Study
ChiCTR2500097585	Prospective, Multi-Center Cohort Study on Deep Cervical Lymphatic-Venous Anastomosis in the Treatment of AD	Kunming Sanbo Brain Hospital	Interventional Study
ChiCTR2500095309	Multi-Center, Prospective Clinical Study on Bilateral Deep Cervical LVA in the Treatment of Moderate to Severe AD	Second Affiliated Hospital of Harbin Medical University	Interventional Study
ChiCTR2400094603	Randomized Controlled Clinical Trial of Deep Cervical Lymphatic-Venous Anastomosis in the Treatment of AD	Zunyi First People’s Hospital	Interventional Study
ChiCTR2400093030	Study on Exploring the Clinical Efficacy of Deep Cervical Lymphatic-Venous Anastomosis in the Treatment of Type 2 Diabetes Mellitus Complicated with Alzheimer’s Disease	Zhengzhou Central Hospital	Observational Study
ChiCTR2400092975	Evaluation of the Efficacy of Comprehensive Diagnosis and Treatment of Alzheimer’s Disease Based on Bilateral Deep Cervical Lymphatic-Venous Anastomosis	Zhengzhou Central Hospital	Interventional Study
ChiCTR2400091764	Study on the Effects of Propofol and Sevoflurane Anesthesia on AD-Related Biomarkers and Behavioral Cognitive Outcomes in AD Patients after Deep Cervical Lymphatic-Venous Anastomosis	Zunyi First People’s Hospital	Interventional Study
ChiCTR2400089883	Single-Center, Prospective, Single-Arm Exploratory Study on Deep Cervical Lymphatic-Venous Anastomosis in Improving Neurological Function in Alzheimer’s Disease Patients	Department of Neurosurgery, First Affiliated Hospital of Army Medical University	Interventional Study
ChiCTR2400084617	Randomized Controlled Clinical Trial of Deep Cervical Lymphatic-Venous Anastomosis and Lymphatic Trunk Lysis in the Treatment of Alzheimer’s Disease	Ninth People’s Hospital Affiliated to Shanghai Jiao Tong University School of Medicine	Interventional Study

AD, Alzheimer’s Disease; LVA, lymphatic-venous anastomosis; fNIRS functional Near-Infrared sSpectroscopy.

In summary, the current clinical implementation of LVA in China vividly illustrates universal ethical challenges arising from introducing invasive neurological interventions in vulnerable populations, such as dementia patients. Analyzing LVA in this broader global context deepens understanding of these ethical concerns and underscores the urgent need for a robust, internationally collaborative ethical and regulatory framework.

## 7 Future directions for prudent breakthroughs

The history of medical innovation is a double-edged sword, reflecting both remarkable successes and cautionary failures. The trajectory of the 20th-century prefrontal lobotomy (Terrier et al., 2019), initially celebrated with a Nobel Prize for its “breakthrough” efficacy but later abandoned due to irreversible damage, contrasts sharply with the century-long evidence-based journey of probiotics (Adams, 2010), which transitioned carefully from hypothesis to clinical validation. These divergent paths offer critical insights for evaluating LVA. To fully realize LVA’s potential and avoid past mistakes, clinical advancement must strictly follow evidence-based medical principles. Only after clearly defining surgical mechanisms, demonstrating robust clinical evidence of efficacy and safety, and establishing comprehensive ethical oversight frameworks should LVA be cautiously integrated into clinical practice.

Future research must prioritize fundamental scientific investigation. Researchers should develop animal models that more accurately reflect human aging processes and AD pathology. Such models could validate LVA’s direct role in clearing intracranial Aβ and tau, quantify intracranial waste clearance after lymphatic-venous anastomosis, and determine whether the benefits are limited to peripheral lymphatic metabolism. Concurrently, LVA’s impact on neuroinflammation and cognitive function should be explored to refine the underlying mechanistic framework. Large animal models (e.g., non-human primates) that closely mimic human cervical lymphatic anatomy should be utilized to evaluate surgical feasibility and long-term anastomotic patency. Postmortem human cadaver studies are necessary to optimize surgical target localization.

Clinical research must be limited to ethically sound, rigorously designed, and strictly managed randomized controlled trials (RCTs), avoiding premature clinical promotion. Such studies should employ multicenter collaboration to increase sample sizes, establish long-term follow-up protocols, and strictly enforce randomization and blinded assessments to clarify true efficacy and long-term safety. Patient selection should adhere to stringent pathological indicators and imaging criteria, focusing particularly on clearly defined cerebral lymphatic drainage disorders to enhance research specificity and generalizability. Surgical techniques and evaluation methods (standardized anastomotic approaches, anastomotic diameters, and postoperative imaging protocols) must be standardized to ensure consistency across studies. Primary clinical endpoints should involve widely validated cognitive assessments, with secondary endpoints incorporating pathological biomarkers, imaging outcomes, and quality-of-life measures.

Technologically, improvements are necessary for intraoperative navigation and localization. Fluorescent dyes with enhanced penetration depth and sensitivity should be developed to improve real-time identification of deep cervical lymphatic-venous structures. Concurrently, robotic-assisted microsurgical techniques should be explored to enhance precision and reduce operative risks. The technical challenges facing LVA are not unique. As highlighted by a comprehensive review on AD surgical interventions (Xie F. et al., 2025), every invasive therapy, including optimized deep brain stimulation, gene therapy crossing the blood-brain barrier, or cerebrospinal fluid shunt devices, faces significant technical complexities. Translational success depends on overcoming these barriers.

Clear ethical guidelines must be established for involving cognitively impaired patients in invasive procedures. Ethical safeguards are essential for this vulnerable population. Although graded informed consent frameworks exist internationally, they must be rigorously enforced and strengthened for high-risk interventions like LVA. Mandatory independent ethics committee reviews, compulsory trial registration, and transparent data reporting should be enforced to protect patients and prevent exaggerated claims of efficacy. Only through rigorous scientific research and robust ethical oversight can the clinical efficacy and safety of LVA for Alzheimer’s disease be accurately assessed.

## 8 Conclusion

In summary, this review has critically assessed the current evidence for LVA in AD. It reveals a promising theoretical framework that remains unsupported by robust clinical evidence for efficacy and long-term safety. Consequently, future development strategies require reconsideration. Rather than rapidly expanding clinical adoption, the path forward should emphasize cautious progression through rigorous, collaborative, phased validation processes. By grounding LVA development in rigorous science, high-quality evidence, and strict ethical standards, researchers can responsibly explore its therapeutic potential, aiming to achieve tangible patient benefits while steadfastly avoiding the mistakes of medical history.
